# Impact of mandatory masking amid the COVID-19 pandemic on outdoor smoking: an interrupted time-series analysis of a 33-month unobtrusive observational study

**DOI:** 10.3389/fpubh.2023.1136621

**Published:** 2023-07-13

**Authors:** Yuying Sun, Yongda Socrates Wu, Yee Tak Derek Cheung, Man Ping Wang, Jianjiu Chen, Lok Tung Leung, Xiaoyu Zhang, Kin Yeung Chak, Tai Hing Lam, Sai Yin Ho

**Affiliations:** ^1^School of Public Health, The University of Hong Kong, Hong Kong, Hong Kong SAR, China; ^2^School of Nursing and Health Studies, Hong Kong Metropolitan University, Hong Kong, Hong Kong SAR, China; ^3^School of Nursing, The University of Hong Kong, Hong Kong, Hong Kong SAR, China; ^4^Department of Epidemiology, Mailman School of Public Health, Columbia University, New York, NY, United States

**Keywords:** COVID-19, face mask, mandatory masking, smoking, observational

## Abstract

**Background:**

Mask-wearing in outdoor public places in Hong Kong was mandated on 29 July 2020, amid the COVID-19 pandemic. We aimed to evaluate the impact of mandatory masking with no exemption for smoking on outdoor smoking.

**Methods:**

We conducted 253 unobtrusive observations at 10 outdoor smoking hotspots in 33 months from July 2019 to March 2022 and counted smokers and non-smoking pedestrians in fixed boundaries. We conducted interrupted time-series analyses on the monthly mean volume of smokers (persons per hour) using generalized linear models. The independent variables were as follows: time since the first observation, implementation of the mask regulation, time since the regulation, seasonality, and waves 1–5 outbreaks. We checked the robustness of the association using the daily mean volume of smokers as the dependent variable. Two sensitivity analyses were conducted to include the hotspot location or the number of all pedestrians as an offset.

**Results:**

Monthly outdoor smoking decreased immediately after the regulation (incidence rate ratio [IRR]: 0.505, 95% confidence interval [CI]: 0.374 to 0.680, *P* < 0.001). Daily smoking analysis and the two sensitivity analyses supported the results. However, monthly outdoor smoking increased by 11% since the regulation (IRR: 1.110, 95% CI: 1.074 to 1.147, *P* < 0.001). An exception was observed at the most severe wave 5 outbreak when monthly outdoor smoking decreased (IRR: 0.415, 95% CI: 0.327 to 0.525, *P* < 0.001).

**Conclusion:**

Outdoor smoking fell immediately after mandatory masking, rebounded to pre-pandemic levels, and decreased again at the most severe wave 5.

## Introduction

In Hong Kong, mass masking was advocated in the early days of the outbreak of COVID-19 (1). Almost 100% voluntary masking was achieved approximately 5 months before masking was made mandatory (2). We had reported that smoking hotspots were typically outdoor non-smoke-free places with rubbish bins for collecting cigarette butts in Hong Kong (3), where the total ban on smoking in all indoor public and workplaces started in 2007 (fixed penalty HK$1,500). Based on unobtrusive observations, we found that smoking clusters involving two or more tobacco users standing closely or talking face-to-face in a circle were common at hotspots (4). Although 96.0% of hotspot tobacco users possessed face masks in the early days of the COVID-19 outbreak, only 67.6% would put them on immediately after smoking (4).

The Hong Kong SAR government implemented mandatory face mask regulation in outdoor public places on 29 July 2020 with a heavy penalty (fixed penalty of HK$5,000; maximum HK$10,000, mandatory face mask indoors started on 23 July 2020) (5). Outdoor masking is exempted for strenuous physical activities and eating during non-outbreak periods, but smoking was expressly not exempted. Mask-wearing amid the pandemic was widely recommended or mandated across countries before December 2020 (6). The mask order issued by the U.S. Center for Disease Control and Prevention also had no exemption for tobacco use (7). The data from our previous publication were collected when the mandatory masking had not commenced (4). It showed that voluntary masking in Hong Kong had been over 95% (4), and there was little room left for further reduction of unmasking. In addition to masking behaviors, the policy would also affect smoking behaviors. As smoking was not exempted from the new policy, outdoor smoking has become illegal since the commencement of the policy. We hypothesized that it would reduce outdoor smoking behaviors immediately and aimed to explore how long the effect could sustain.

Some studies have explored the effect of the COVID-19 pandemic on smoking. Some studies showed that more smokers increased the use of tobacco products (8, 9), while some showed more decrease in use (10, 11). While most social distancing measures decreased the use of tobacco products, unemployment and anxiety may increase the use of tobacco products (12, 13). Masking is an impact factor that might cause the reduction of smoking or quitting (14). We evaluated the impact of mandatory masking on outdoor smoking through a 33-month unobtrusive observational study in Hong Kong, adjusting for the five waves of the COVID-19 outbreak and seasonality.

## Materials and methods

### Study design, setting, and population

We conducted an unobtrusive observational study to monitor outdoor smoking in July 2019 by recording the volume (persons per hour) of smoking and non-smoking pedestrians. Details of the methods have been reported (4). Ethical approval was granted by the Institutional Review Board of the University of Hong Kong/Hospital Authority Hong Kong West Cluster (reference number: UW 19-169). Based on our previous studies and pilot observations in different districts (3, 15), we selected 10 hotspots with the largest number of pedestrians and tobacco users. The boundaries of each hotspot were delineated by fixed structures (e.g., poles, walls, and curbs) or environmental markings. The size of each hotspot was approximately 20 m^2^. People using any tobacco products at the hotspots were deemed tobacco users.

The observations were conducted around noon (11 am−2 pm) or in the afternoon (3 pm−6 pm), lasting 2 or 3 h. A total of 37 observers were trained. The interrater reliability has been reported, showing moderate to excellent reliability of the observations on smokers (4). The observations were conducted unobtrusively approximately 10 m away. The observers documented each tobacco user's sex (male and female), age group (adolescents aged under 21 years, young adults aged 21–40 years, middle-aged 41–60 years, and older people above 60 years), types of tobacco products used (cigarettes, heated tobacco products [HTPs], electronic cigarettes [e-cigarettes], and others), duration of stay (passer-by, <1 min, and ≥ 1 min), and the number of cigarettes consumed (1 cigarette was equivalent to “1 HTP heat stick” or “15 puffs or 10 min of e-cigarette use”) on a paper form. Smoking clusters referred to two or more tobacco users who stayed close, often facing each other and chatting.

### Data

Interrupted time-series (ITS) analysis is a valuable study designed for evaluating the effectiveness of population-based health interventions or national public health legislation (16). To conduct ITS analysis, we used two dependent variables. One was the daily volume of smokers (daily outdoor smoking) at 10 different smoking hotspots. As the frequency of our observations at each hotspot was roughly monthly, we summed up daily records to the monthly mean volume of smokers (monthly outdoor smoking) as another dependent variable. We conducted 253 hotspot observations in 232 days out of 1002 days (33 months) from July 2019 to March 2021. We did not conduct any observation in March 2020, given the uncertain risk of COVID-19 infection in the early days of the outbreak. Therefore, we had 32 monthly data points and 232 daily data points. The sample size was 32 and 232 for respective analyses. There was no gold standard in the minimum number of time points required for conducting an ITS, while a scoping review showed that a minimum of eight time points per period is needed (17). The sample size per time point also matters, although there needs to be more guidance on this (17). We observed for over 1 hour at each hotspot and observed more than 200 smokers at some hotspot sites. Observing longer time and more smokers might reduce the variability and outliers.

The data structure for analysis was organized following the requirements of conducting a standard time-series analysis (16, 18). Both the level change (the change immediately after implementing the regulation) and slope change (the change in slope after implementing the regulation) were calculated (16, 18). The intercept was the volume of smokers on the first day or the first month. Time was a continuous variable indicating the day (from 1 to 1,002) or month (from 1 to 33) from the start of the observation period. The intervention, our variable of interest, was implementing the mask regulation. It was a dichotomous variable representing the status before (coded as 0) and after (coded as 1) the regulation from month 14 or day 392. Time since the intervention was a continuous variable counting the number of months or days after the regulation at time t, coded 0 before the regulation, and “time-13 months” or “time-391 days” after the regulation. Because Hong Kong's climate is sub-tropical, we coded seasonality as a dichotomous variable to indicate the observation season in winter or other seasons (December to February were coded as winter; additional months as other seasons) (19).

As the different waves of the outbreak might also influence outdoor smoking behaviors, we defined four outbreak periods for controlling potential bias using dichotomous variables (coded 1 if the observed date fell into the period and 0 if it was not in the period): (1) wave 1 and wave 2 (February–April 2020), (2) wave 3 (July–September 2020), (3) wave 4 (November 2020–April 2021), and (4) wave 5 (January–March 2022) (20). The anti-epidemic measures, such as limiting gathering up to two or four persons and working from home, implemented during each wave of the outbreak, were not adjusted to avoid repetition.

### Statistical analysis

The dependent variable in the primary analysis was monthly outdoor smoking (*n* = 32 time points), which was count data and assumed to follow a Poisson distribution. We also checked the robustness of the association by using daily outdoor smoking as the dependent variable (*n* = 232 time points). We assessed non-stationarity by the augmented Dickey–Fuller test that indicates a non-stationary time series (monthly data: Dickey–Fuller statistic = -2.06, *P* = 0.55; daily data: Dickey–Fuller statistic = -1.87, *P* = 0.63). We conducted Durbin Watson test to check the autocorrelation of the data. The autocorrelation was double-checked by examining the autocorrelation function plots. Both results indicated no serious autocorrelation (monthly data D-W statistic = 1.98, *P* = 0.21; daily data D-W statistic = 1.67, *P* = 0.41; Supplementary Figure 1 shows the residual plots of the dependent variables). Therefore, we used generalized linear models to fit the data. The ‘intervention' of interest in this study was implementing mask regulation. The independent variables also included time since the first observation, time since the intervention, seasonality, and waves 1–2, 3, 4, and −5 outbreaks. We assumed that the target population size (the denominator: the number of smokers in the district or the whole territory) was stable during the observation period, so we did not include offset in the primary analysis. The incidence rate ratio (IRR) and 95% confidence interval (CI) were calculated. A *P* < 0.05 for two-tailed tests indicated statistical significance.

We undertook two sensitivity analyses. First, we repeated the ITS analysis by adding a dichotomous variable of hotspot location [1 = business district, mainly (Admiralty, Hong Kong Station, Causeway Bay, Sheung Wan, Tsim Sha Tsui, Kowloon Tong); 0 = business and residential district [Mong Kok, Kwun Tong, Tsuen Wan, Kwai Fong] for the daily-based dependent variable. Second, we repeated the ITS analysis by including the number of all pedestrians (sum of smoking and non-smoking pedestrians) as an offset. As the number of all pedestrians already reflected the severity of COVID-19, outbreak waves were excluded from this sensitivity analysis.

## Results

Table 1 shows that of the 31,273 observed tobacco users, most were men (73.5%), young and middle-aged (93.9%), cigarette users (92.1%), stayed at the smoking hotspot for 1 min or longer (88.8%), smoked alone (81.6%), and consumed only one cigarette or equivalent (97.3%).

**Table 1:** Characteristics of 31,273 tobacco users at smoking hotspots.

**Features**	**Categories**	**Total**
Sex	Male	22,996 (73.5)
	Female	8,277 (26.5)
Age, years	Adolescents aged under 21	218 (0.7)
	Young adults 21–40	18,218 (58.3)
	Middle-aged 41–60	11,141 (35.6)
	Older people above 60	1,666 (5.3)
	Missing	30 (0.1)
Type of tobacco products used^a^	Cigarettes	28,814 (92.1)
	E-cigarettes	960 (3.1)
	Heated tobacco products	1,448 (4.6)
	Missing	51 (0.2)
Duration of stay	Passer-by	1,693 (5.4)
	<1 min	1,736 (5.6)
	≥ 1 min	27,767 (88.8)
	Missing	77 (0.2)
Smoking cluster or alone	Smoked alone	25,519 (81.6)
	Two or more	5,754 (18.4)
Number of cigarettes consumed	One	30,437 (97.3)
	Two or more	836 (2.7)

a 1 cigarette equals “1 heated tobacco product heat stick” or “15 puffs or 10 min of e-cigarette use.”

**Figure 1:**
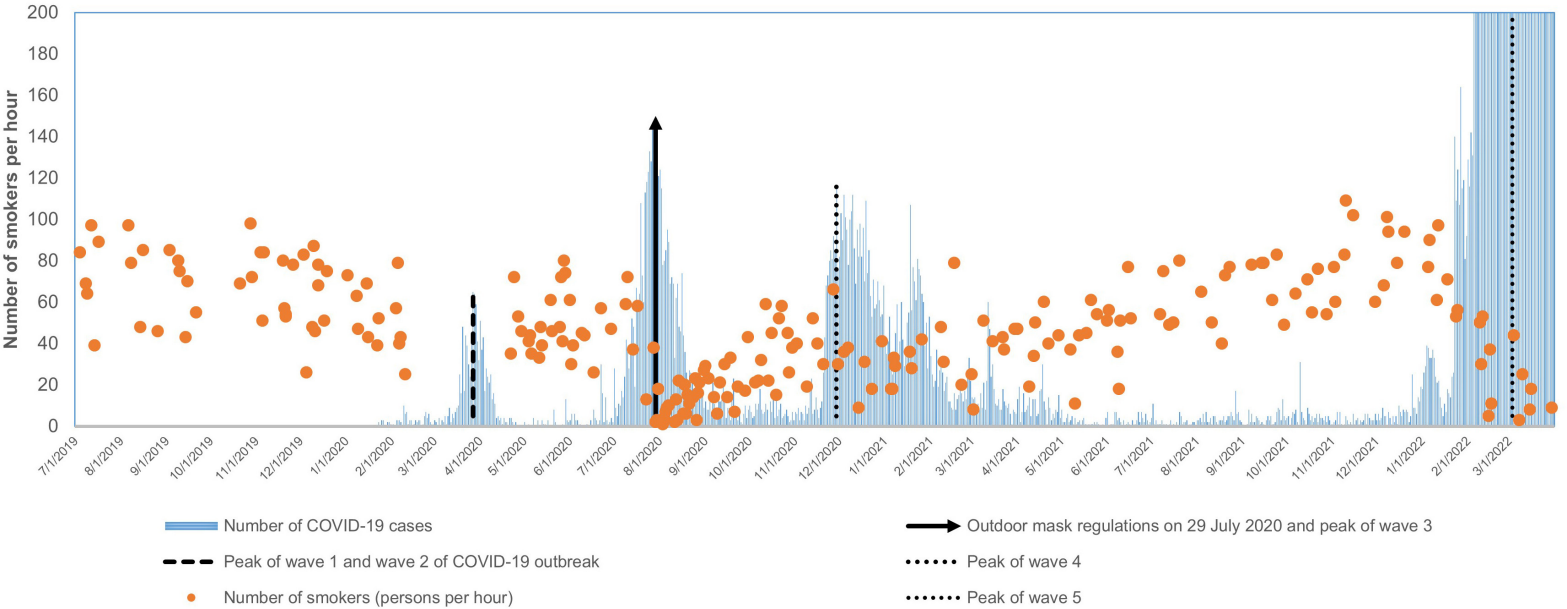
The daily volume of tobacco users (persons per hour) in the study period and the number of COVID-19 cases since the outbreak. The y-axis could not show the number of COVID-19 cases from 5 February to 31 March 2022 due to the large numbers (13,099 cases per day on average) relative to the volume of tobacco users. The peak of waves 1 and 2: 65 COVID-19 cases on 27 March 2020. The peak of wave 3: 149 cases on 30 July 2020. The peak of wave 4: 115 cases on 29 November 2020. The peak of wave 5: 76,991 cases on 3 March 2022.

The volume of smokers was 84 persons per hour on the first day of observation. The mean volume was 74 and 47 persons per hour in the first month and over the observation period, respectively. Figure 1 shows daily outdoor smoking and the number of COVID-19 cases. Supplementary Figure 2 shows the number of smokers at 10 hotspot sites. The pattern in the change of smokers at 7 (Hong Kong Station, Causeway Bay, Sheung Wan, Kwun Tong, Tsuen Wan, Kwai Fong, Kowloon Tong [since the first observation date was 6 February 2020, the decreasing trend before the mask regulation was not clear]) out of 10 sites was similar to the overall changes. Admiralty, Tsim Sha Tsui, and Mong Kok did not show a clear increasing trend after the mask regulation. Table 2 shows the results of the ITS analysis on monthly and daily outdoor smoking. The primary analysis showed an underlying decreasing trend of both monthly (IRR: 0.966, 95% CI: 0.945 to 0.988, *P* = 0.003) and daily (IRR: 0.9988, 0.9985 to 0.9991, *P* < 0.001) outdoor smoking. On top of this underlying trend, we found a significant decrease in both monthly (IRR: 0.505, 0.374 to 0.680, *P* < 0.001) and daily (IRR: 0.531, 0.477 to 0.591, *P* < 0.001) outdoor smoking immediately after the regulation. However, outdoor smoking soon increased (monthly IRR: 1.110, 1.074 to 1.147, *P* < 0.001; daily IRR: 1.003, 1.003 to 1.004, *P* < 0.001). An exception was the most and much more severe wave 5 outbreak, when outdoor smoking decreased (monthly IRR: 0.415, 0.327 to 0.525, *P* < 0.001; daily IRR: 0.447, 0.405 to 0.494, *P* < 0.001). No seasonal effect was found (monthly IRR: 1.039, 0.923 to 1.168, *P* = 0.52; daily IRR: 1.034, 0.983 to 1.086, *P* = 0.19). The associations between the implementation of the regulation and the outcomes were robust as supported by the two sensitivity analyses.

**Table 2:** The incidence rate ratio for the impact of mask regulation on monthly and daily volume of smokers at smoking hotspots.

**IRR (95% CI)**	**Monthly volume of smokers (*n =* 32)**	**Daily volume of smokers (*n =* 232)**
**Main analysis**		
Time	0.966 (0.945, 0.988)^**^	0.9988 (0.9985, 0.9991)^***^
Mask regulation	0.505 (0.374, 0.680)^***^	0.531 (0.477, 0.591)^***^
Time since the intervention	1.110 (1.074, 1.147)^***^	1.003 (1.003, 1.004)^***^
Seasonal effect (winter)	1.039 (0.923, 1.168)	1.034 (0.983, 1.086)
Wave 1 and wave 2 outbreaks	0.867 (0.688, 1.085)	0.890 (0.802, 0.985)^*^
Wave 3 outbreak	0.768 (0.579, 1.011)	0.672 (0.607, 0.743)^***^
Wave 4 outbreak	0.909 (0.738, 1.118)	0.945 (0.874, 1.021)
Wave 5 outbreak	0.415 (0.327, 0.525)^***^	0.447 (0.405, 0.494)^***^
**Sensitivity analysis 1 (add location)**		
Time	-	0.9987 (0.9984, 0.999)^***^
Mask regulation	-	0.540 (0.485, 0.601)^***^
Time since the intervention	-	1.004 (1.003, 1.004)^***^
Seasonal effect (winter)	-	1.031 (0.981, 1.084)
Wave 1 and wave 2 outbreaks	-	0.920 (0.829, 1.019)
Wave 3 outbreak	-	0.683 (0.617, 0.756)^***^
Wave 4 outbreak	-	0.956 (0.884, 1.034)
Wave 5 outbreak	-	0.449 (0.407, 0.496)^***^
Location (business district mainly)	-	1.148 (1.099, 1.198)^***^
**Sensitivity analysis 2 (include offset and exclude outbreak waves)**		
Time	1.0004 (0.9812, 1.0198)	1.0001 (0.9999, 1.0004)
Mask regulation	0.551 (0.439, 0.692)^***^	0.631 (0.575, 0.691)^***^
Time since the intervention	1.057 (1.033, 1.082)^***^	1.001 (1.001, 1.002)^***^
Seasonal effect (winter)	0.857 (0.766, 0.957)^**^	0.974 (0.929, 1.022)

IRR, incidence rate ratio; CI, confidence interval.

* *P* < 0.05,

** *P* < 0.01,

*** *P* < 0.001.

## Discussion

We have first shown the impact of mandatory masking with no exemption for smoking on outdoor smoking. The mask regulation in Hong Kong was associated with an immediate drop in outdoor smoking at hotspots after adjusting for the impact of the COVID-19 outbreak and seasonality. Regarding mask regulation's impact on outdoor smoking, we have shown similar results using monthly data (smaller sample size) and daily data (bigger sample size). The results should be robust. Emphasis on no exemption for smoking in the early days (5) and fixed penalty tickets issued during enforcement might have deterred many smokers initially. However, the effects were short-lived as outdoor smoking soon rebounded.

The rebound probably reflected the weak enforcement of the mask and smoking regulations and the scarcity of mass media reports on enforcement. With a population of 7.5 million and 691,500 smokers (21), the daily penalty tickets issued in Hong Kong ranged from 5 to 20 from July 2020 to January 2022 for the violation of the mask regulation^1^ and from 14 to 23 during July 2019 to March 2022 for the violation of the Smoking (Public Health) Ordinance (22). The daily number of penalty cases for both regulations was low, given that the enforcement should have covered the whole city and not just hotspots. We conducted 253 observations but observed <10 occurences of enforcement actions at smoking hotspots. The media reported penalty cases in the early days of the mandatory masking and during the wave 5 outbreak (23–25), but reports were few. The rebounds after the regulation probably also indicated pandemic fatigue (26) in tobacco use. Such rebound might have been suppressed if enforcement had been strengthened with wide publicity earlier.

The mask regulation was not designed as a tobacco control measure but might have served to reduce smoking incidentally. The prevalence of daily conventional cigarette smokers in Hong Kong declined from 10.2% in 2019 to 9.5% in 2021 (27). Our previous qualitative study showed a decrease in tobacco use in one-third of participants due to fear of being fined for taking off the mask in public places, and some were thus motivated to quit smoking (14). It indicated that the inconvenience of outdoor smoking caused by mandatory masking might result in quitting smoking. In our previous report, by synthesizing four surveys in Hong Kong in the early stage of the outbreak, 51.9% of smokers decreased tobacco use outdoors, while 22.1% increased use at home (28). We also conducted a community-based telephone survey between the second and third waves of the COVID-19 pandemic in Hong Kong. We found that avoiding smoking on the street (prevalence: 58.9%) and reducing going out to buy cigarettes (33.5%) were associated with more quit attempts and smoking reduction (12). However, future research needs to explore the change in the overall consumption of tobacco products.

We conducted observations in three districts of Hong Kong, which provides an overall picture of the change in smokers' outdoor smoking behaviors. Instead of one single survey, our study reflected the dynamic evolution of outdoor smoking over a long period (33 months) covering both pre-pandemic and since-pandemic periods. Since the WHO announced an end to COVID-19 as a global health emergency on 5 May 2023 (29), the effect of the pandemic on outdoor smoking behaviors might have disappeared. However, continuous monitoring of hotspot smoking is likely to be a valuable method to understand the impact of various new policies on smoking behaviors instantly, such as banning alternative products and expanding smoke-free areas. Such observation methods could also be used in other public health topics where unobtrusive observations could be conducted.

Our study had some limitations. Because we purposefully selected hotspots with the largest number of pedestrians in Hong Kong, typically in busy districts, they might only represent some tobacco users in some hotspots and the territory. However, the association between mask regulation and outdoor smoking could be more readily observed there. Second, we included several factors to adjust for the impact of the pandemic and seasonality, but there might be other unmeasured time-varying confounders. Since we have conducted several sensitivity analyses, the effect of such confounders, if any, was likely insignificant.

## Conclusion

In this 33-month unobtrusive observational study, we found that outdoor smoking at smoking hotspots fell immediately after mandatory masking in Hong Kong, rebounded to pre-pandemic levels probably because of weak law enforcement, but decreased again at the most severe wave 5 of the COVID-19 outbreak.

## Data availability statement

The raw data supporting the conclusions of this article will be made available by the authors, without undue reservation.

## Ethics statement

The studies involving human participants were reviewed and approved by the Institutional Review Board of the University of Hong Kong/Hospital Authority Hong Kong West Cluster (reference number: UW 19-169). Written informed consent for participation was not required for this study in accordance with the national legislation and the institutional requirements.

## Author contributions

YTDC, MPW, JC, LTL, THL, and SYH: funding acquisition. YS and YSW: methodology, validation, and data curation. YS, XZ, KYC, YTDC, and SYH: data collection, project administration, and supervision. YS: writing—original draft preparation. All authors contributed to the study conceptualization, writing—review and editing, and approved the final version of the manuscript.

## Footnotes

^1^Health Bureau of the Government of the Hong Kong Special Administrative Region. *The Numbers of Inspections, Warnings, Fixed Penalty Tickets and Prosecutions of the Prevention and Control of Disease (Prohibition on Group Gathering) Regulation (Cap. 599g)*. (2022).
