# Supplementary material for: Impact of mandatory masking amid the COVID-19 pandemic on outdoor smoking: an interrupted time-series analysis of a 33-month unobtrusive observational study
Source: Front Public Health. 2023 Jul 13;11:1136621. doi: 10.3389/fpubh.2023.1136621 (PMC10372439; doi:10.3389/fpubh.2023.1136621)

Supplementary Material

Impact of mandatory masking amid the COVID-19 pandemic on outdoor smoking: an interrupted time series analysis of a 33-month unobtrusive observational study

**Yuying Sun^1,2^, Yongda Socrates Wu^3^, Yee Tak Derek Cheung^3,*^, Man Ping Wang^3^, Jianjiu Chen^1,4^, Lok Tung Leung^1^, Xiaoyu Zhang^1^, Kin Yeung Chak^1,3^, Tai Hing Lam^1^, Sai Yin Ho^1^**

*** Correspondence:** Yee Tak Derek Cheung: derekcheung@hku.hk

## Supplementary Figures

**Supplementary Figure 1. Residuals plots of the dependent variables in the main analysis.**


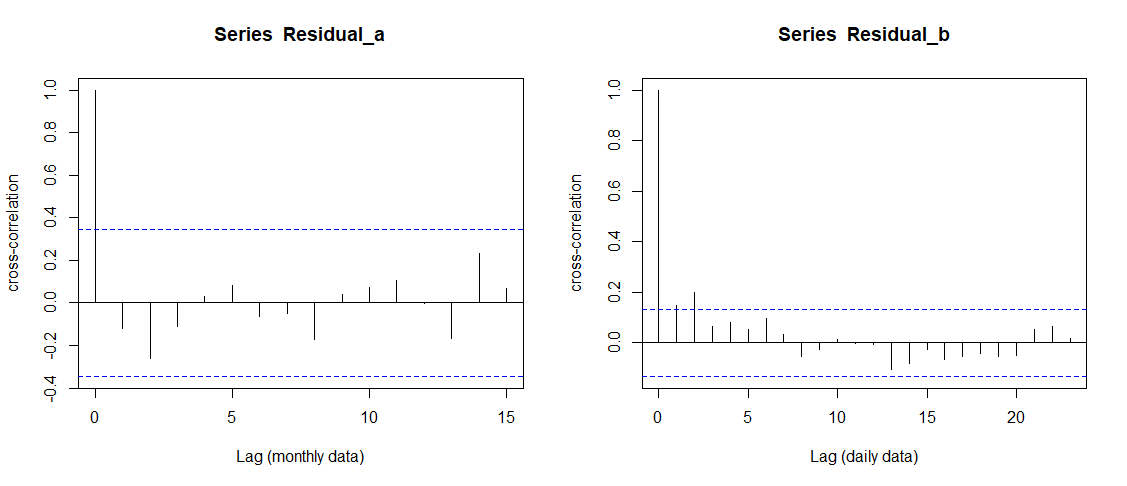


Residual_a: monthly data; Residual_b: daily data.

**Supplementary Figure 2.** Number of smokers per hour at 10 smoking hotspots.


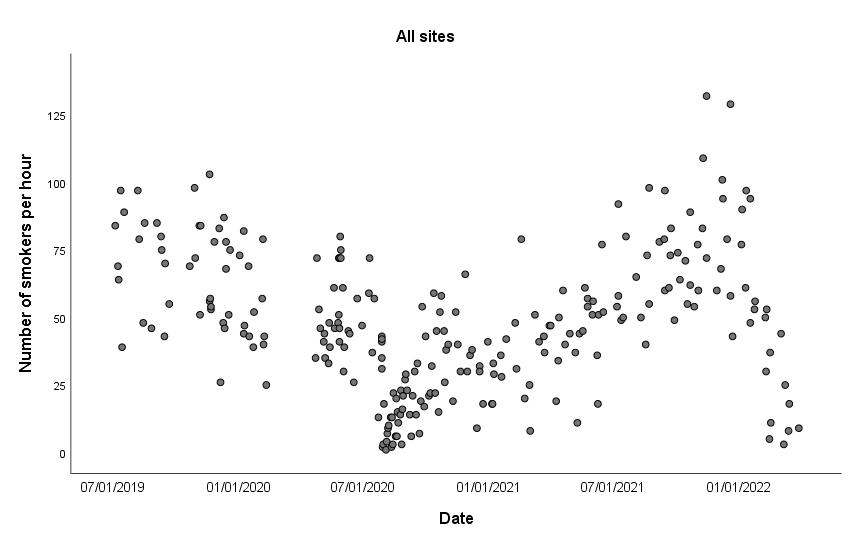


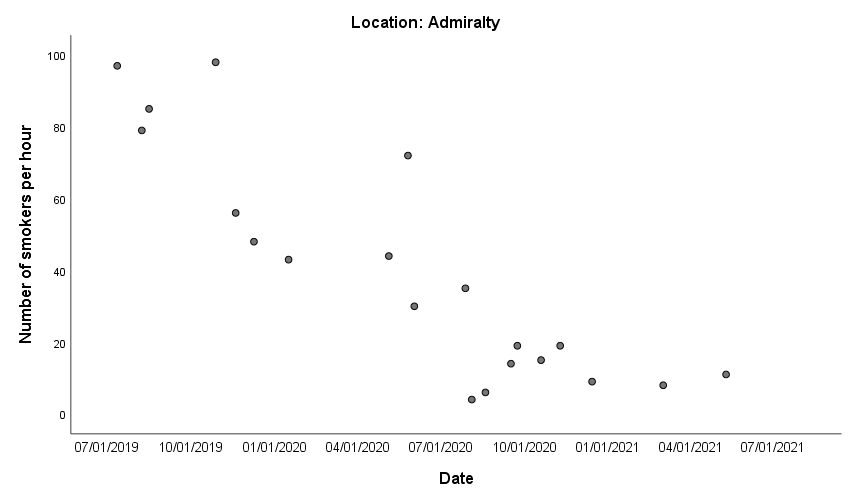

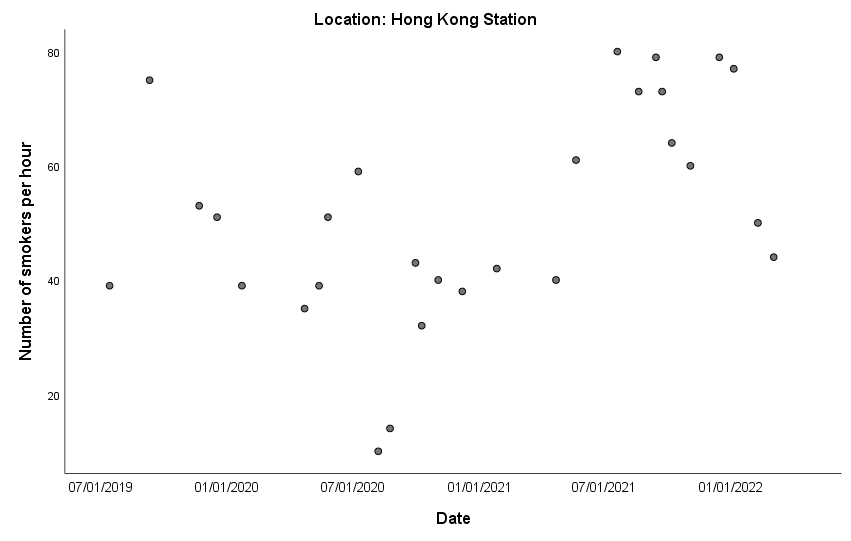


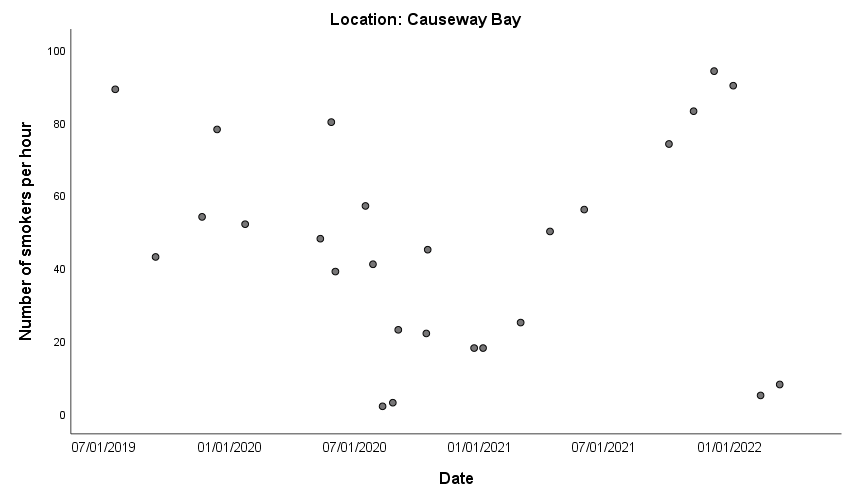

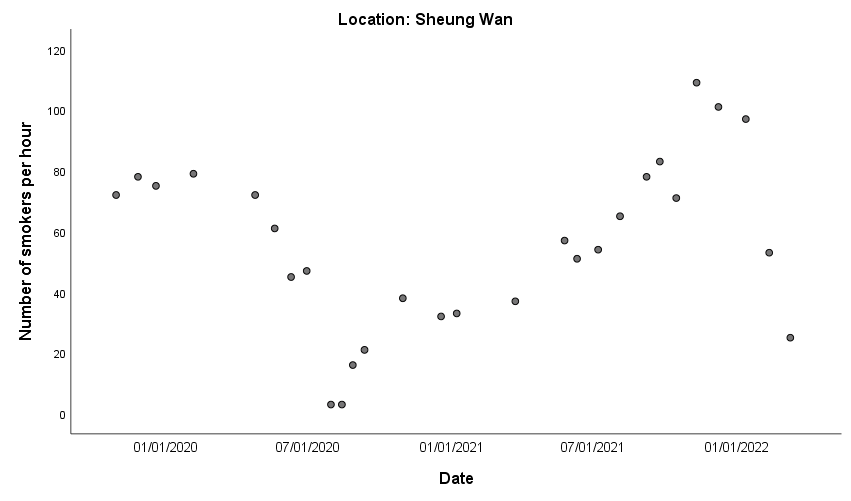


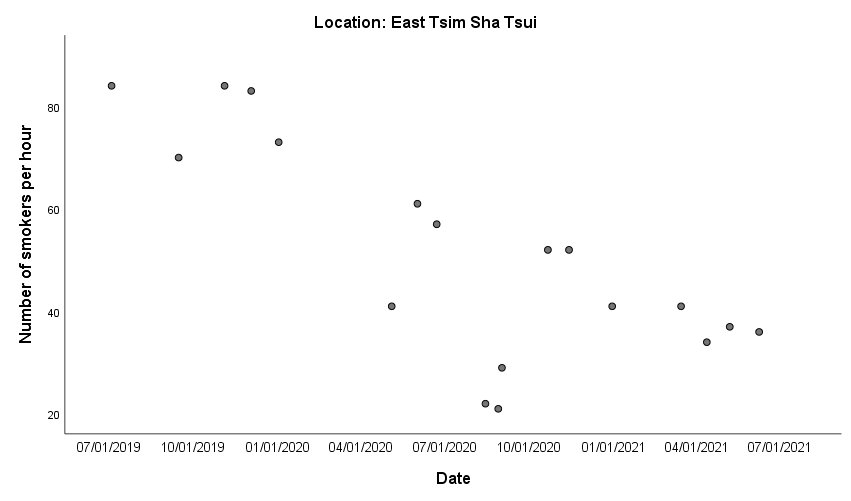

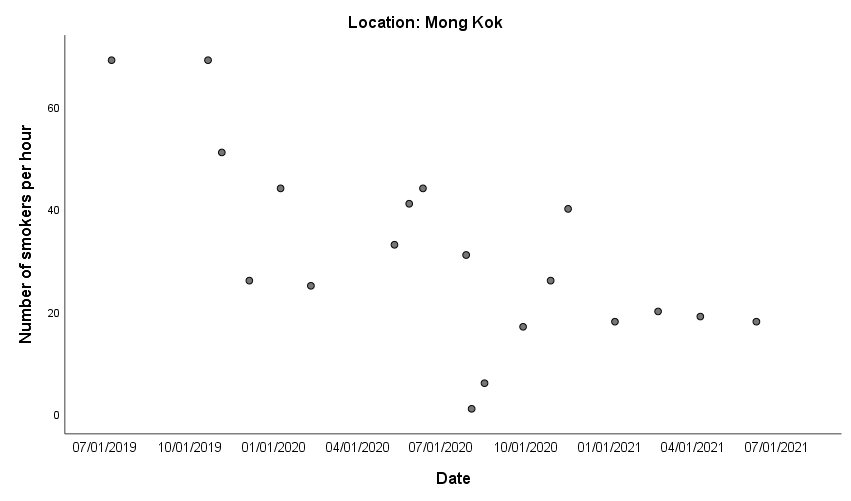


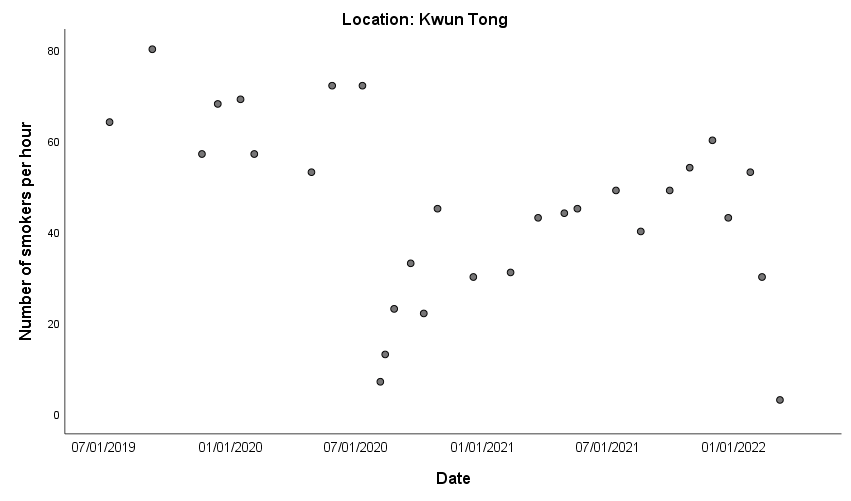

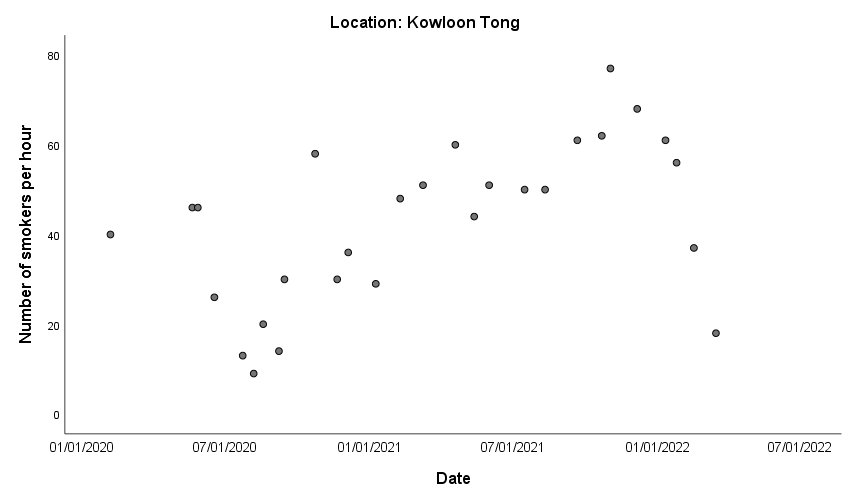


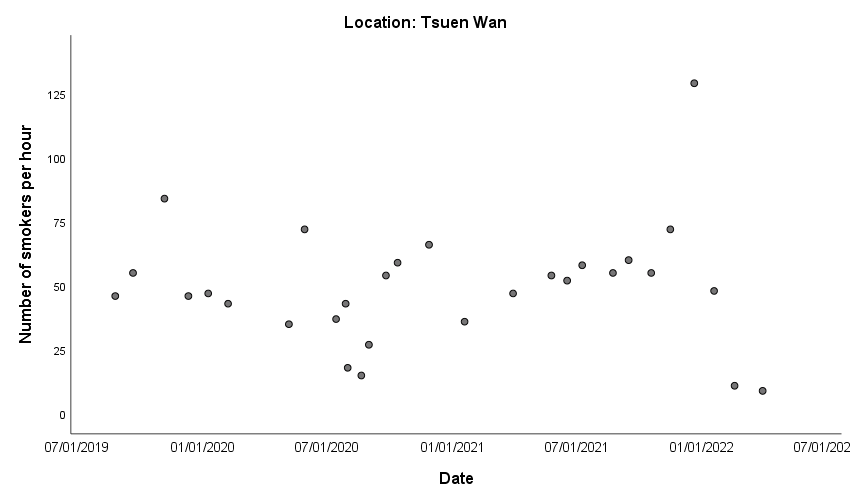

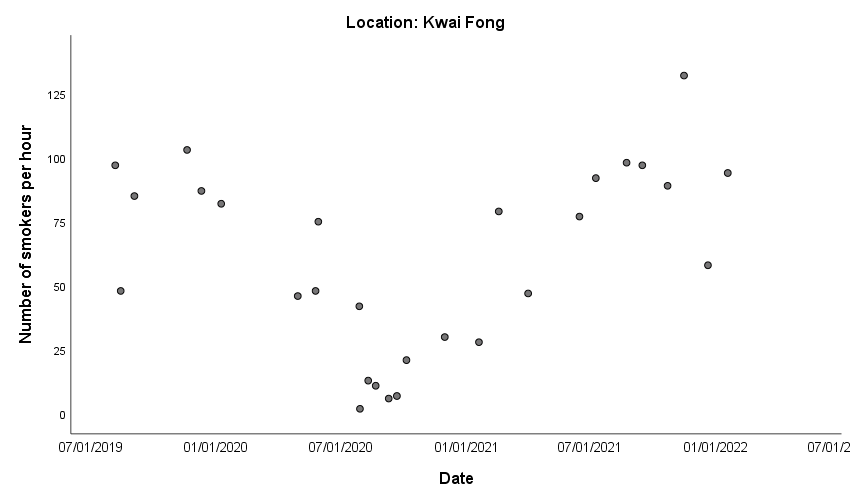

Supplement: Supplementary file 1 [file Table_1.DOCX]
